# Effect of Cold Rolling on the Phase Transformation Kinetics of an Al_0.5_CoCrFeNi High-Entropy Alloy

**DOI:** 10.3390/e20120917

**Published:** 2018-11-30

**Authors:** Jun Wang, Haoxue Yang, Tong Guo, Jiaxiang Wang, William Yi Wang, Jinshan Li

**Affiliations:** State Key Laboratory of Solidification Processing, Northwestern Polytechnical University, Xi’an 710072, China

**Keywords:** high-entropy alloy, phase transformation, kinetics, deformation, thermal expansion

## Abstract

The solid state phase transformation kinetics of as-cast and cold rolling deformed Al_0.5_CoCrFeNi high-entropy alloys have been investigated by the thermal expansion method. The phase transformed volume fractions are determined from the thermal expansion curve using the lever rule method, and the deformed sample exhibits a much higher transformation rate. Two kinetic parameters, activation energy (*E*) and kinetic exponent (*n*) are determined using Kissinger– Akahira–Sunose (KAS) and Johnson–Mehl–Avrami (JMA) method, respectively. Results show that a pre-deformed sample shows a much lower activation energy and higher kinetic exponent compared with the as-cast sample, which are interpreted based on the deformation induced defects that can promote the nucleation and growth process during phase transformation.

## 1. Introduction

High-entropy alloys (HEAs) are a new class of alloys designed based on a unique alloy concept that have multi alloy components (normally five or more principal elements) with equal or near-equal atomic composition [1,2]. HEAs are mainly solid solution based alloys, which own very attractive properties, like high strength and hardness, high fracture toughness, excellent corrosion resistance and unique physical properties, making them the potential engineering materials in many industry areas [3,4,5,6,7].

Thermal induced phase transformations are one of the main transitions inside metallic materials which can be used to control the final phases of the material, and thus to tune the properties. In order to know the details of the phase transformation processes, one of the useful methods is to study the phase transformation kinetics, which can give a lot of useful information like phase transformation speed and transition mode [8,9]. However, until now, there have only been very few papers referring to the kinetic analysis of high-entropy alloys.

The as-cast HEAs always exhibit coarse grains especially for big ingots. In order to obtain fine equiaxed grains, methods like cold rolling or forging are incorporated during the treating process [10,11]. Whether this kind of treatment can affect the subsequent phase transformation process is still unclear for HEAs.

Al*_x_*CoCrFeNi (0 ≤ *x* ≤ 2) HEAs are one of the alloy systems that intrigue the research interest due to many aspects. First, this alloy system owns very good mechanical [12,13,14,15] and physical properties [16,17,18]. Second, with the increasing Al content, the main phase of the alloy moves from pure face-centered-cubic (FCC) to FCC + pure body-centered-cubic (BCC), and pure BCC phase, making the mechanical and physical properties adjustable [16,17,18,19,20,21,22,23,24,25]. Thus, the microstructure, phases and properties of this alloy system have been intensively investigated. In this study, a binary phase (FCC + BCC) Al_0.5_CoCrFeNi HEA with a balanced strength and plasticity are chosen to investigate the effect of cold rolling on the subsequent solid state phase transformation kinetics. 

## 2. Experimental Procedures

The ingots with a nominal composition of Al_0.5_CoCrFeNi were prepared by melting pure elements (at least 99.95 wt.%) in a vacuum induction-melting furnace. The furnace is firstly heated to 500 °C and held for 2 h to remove the water vapor, and vacuumed to below 10 Pa. Then pure argon is backfilled to expel the rest of air until the vacuum goes back to standard atmospheric pressure. This process is repeated three times in order to gain an oxygen-free environment as much as possible. Afterwards, melting and casting is performed with the protection of high purity argon and the alloy is heated at about 1550 °C for 15 min. Approximately 15 kg ingot is produced by casting the melt into a steel crucible with a height of 180 mm, upper inner diameter of 140 mm and bottom inner diameter of 130 mm.

Samples with the size of 8 × 20 × 70 mm^3^ are taken from the center of the ingot. Then, the sample is cold rolled to a thickness reduction of 20%. Then, samples were machined to Ф 6 × 25 mm for thermal expansion measurement both from as-cast and cold rolled plates. The thermal expansion curves were tested using a Netzsch^®^ DIL-402C dilatometer (Selb, Germany) under the protection of argon with constant heating rate of 4 K/min, 6 K/min, 8 K/min and 10 K/min.

The microstructure and phases were characterized by scanning electron microscope (SEM, TESCAN MIRA3 XMU (Brno, Czech Republic), and the working distance and the energy of beam used are 15 mm and 20 kV, respectively) and X-ray diffractometer (XRD, DX 2700 (Dandong, China), and the voltage and current used during measurement are 40 kV and 30 mA, respectively), respectively. Uniaxial tensile tests are carried out with a MTS SANS CMT5105 (Shenzhen, China) mechanical tester at the strain rate of 10^−3^ s^−1^ and the specimens are prepared along the rolling direction (RD) direction.

## 3. Results and Discussion

### 3.1. Microstructure

**Figure 1:**
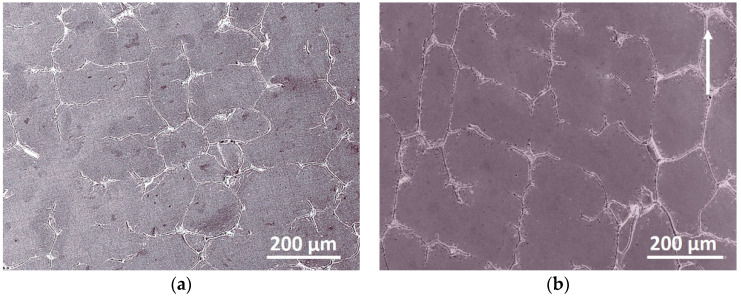
The microstructure of the Al_0.5_CoCrFeNi high-entropy alloys at (**a**) as-cast and (**b**) 20% cold rolled condition. The arrow represents the rolling direction.

**Figure 2:**
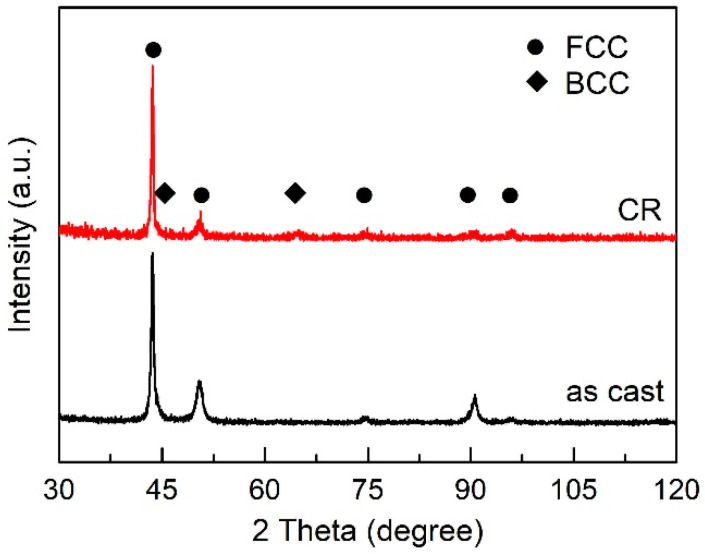
The XRD patterns of Al_0.5_CoCrFeNi high-entropy alloy at as-cast and 20% cold rolled condition.

The microstructure and XRD patterns of Al_0.5_CoCrFeNi high-entropy alloys at as-cast and 20% cold rolled (CR) condition are shown in Figure 1 and Figure 2. Except for the grain after cold rolling showing a very small amount elongation along the rolling direction, the as-cast and cold rolled sample show quite similar microstructure (Figure 1). Both the samples are mainly FCC phase together with a small amount of BCC phase (Figure 2), indicating 20% cold rolling has no significant effect on the microstructure and phase transition of the Al_0.5_CoCrFeNi high-entropy alloy.

### 3.2. Transformed Volume Fraction

**Figure 3:**
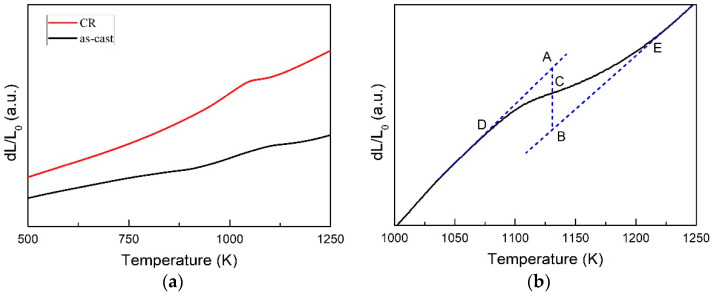
(**a**) thermal expansion curves of Al_0.5_CoCrFeNi high-entropy alloy measured at as-cast and 20% cold rolled condition at heating rate of 10 K/min; (**b**) an example of as-cast sample shows how to calculate the phase transformed volume fraction as function of temperature.

Figure 3a shows the thermal expansion curves of Al_0.5_CoCrFeNi HEA at as-cast and 20% CR condition measured at the heating rate of 10 K/min. There is a peak around 1100 K for both curves indicating a phase transition exist, which is proved to be an FCC-BCC phase transition [8]. The first step for phase transition kinetic analysis is to determine the phase transformed volume fraction (*f*) as a function of temperature or time. Figure 3b shows how to calculate the phase transformed volume as a function of temperature with lever rule method using thermal expansion curves shown in Figure 3a. The lines AD and BE can be obtained by extending the linear expansion section of the thermal expansion curve. Then, the point C is the intersection of AC and thermal expansion curve (*f* = 0 when C is at point D and *f* = 100% when C is point E). Finally, the theoretical volume fraction of phase transition can be calculated using the simple lever rule method assuming the volume changes of phase transition is in proportion to the length variations, which is expressed in Equation (1) [26]:

(1) $$f=\frac{\left|AC\right|}{\left|AB\right|},$$

where *f* is the volume fraction, and *|AC|* and *|AB|* are the length of the dashed line shown in Figure 3b, indicating the instant thermal expansion length and overall length, respectively.

**Figure 4:**
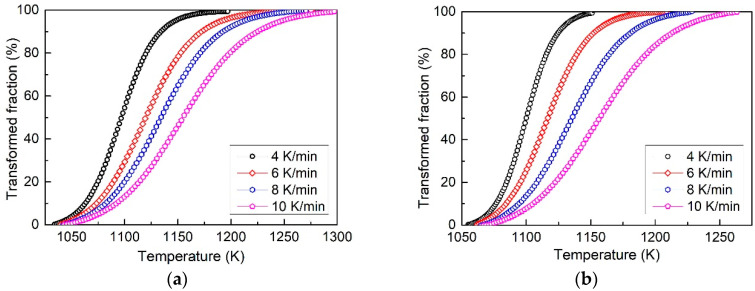
The phase transformed volume fraction as a function of temperature curves of (**a**) as-cast and (**b**) 20% cold rolled Al_0.5_CoCrFeNi high-entropy alloy.

Figure 4 shows the transformed volume fraction as a function of temperature at different heating rates for the as-cast (Figure 4a) and 20% CR (Figure 4b) samples. All of the *f–T* curves show the similar “S” type trend, indicating that the transformation is controlled by a typical nucleation-growth mechanism [27]. Compared with the as-cast sample, it can be seen from Figure 4 that the phase transition of cold rolled sample starts at a much higher temperature (1033 K and 1055 K at 4 K/min for as-cast and cold rolled samples, respectively) but ends at a much lower temperature (1195 K and 1151 K at 4 K/min for as-cast and cold rolled samples, respectively), indicating a much higher transformation rate when the sample is deformed.

### 3.3. Activation Energy

Activation energy (*E*) and kinetic exponent (*n*) are the two most important kinetic parameters. Activation energy is the parameter that can assess phase transition energy barrier. The Kissinger– Akahira–Sunose (KAS) method [28] and Friedman method [29] can be used for the determination of activation energies. Here, we use KAS method, which has the following form [28]:

(2) $$\ln\left(\frac{T^{2}}{\varphi }\right)=-C+\frac{E}{RT},$$

where *T* is temperature at certain transformed volume fraction, *ϕ* is the heating rate, *E* is the activation energy, *C* is a constant and *R* is the molar gas constant. Assuming the activation energy is the same at the same transformed fraction at different heating rates, then, by linear regression between ln*(T^2^/ϕ)* and *1/T* using the data taken from Figure 4, the activation energy can be directly determined by the slope of the curve.

**Figure 5:**
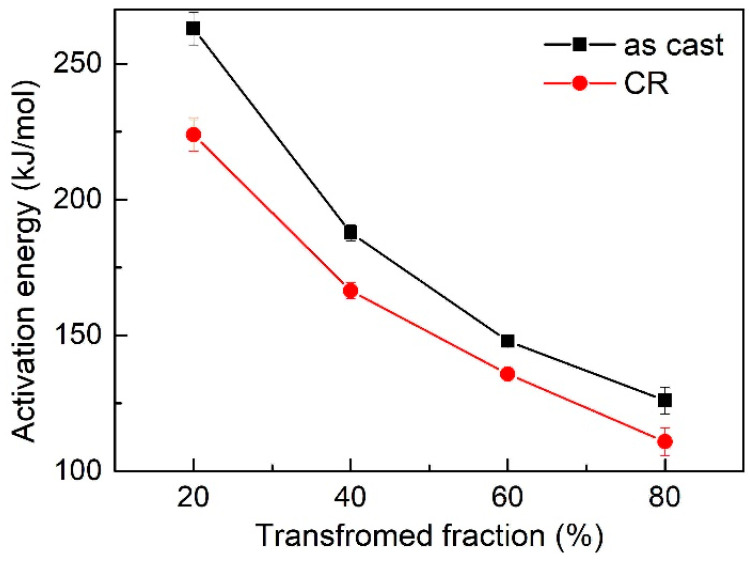
The activation energies at different phase transformed volume fractions for as-cast and cold rolled Al_0.5_CoCrFeNi high-entropy alloys calculated using the KAS method.

The determined activation energy *E* for as cast and 20% CR Al_0.5_CoCrFeNi HEA at four different transformed volume fractions, *f* = 0.2, 0.4, 0.6 and 0.8 are shown in Figure 5. The activation energies of as-cast and 20% CR Al_0.5_CoCrFeNi HEA show the same decreasing trend with the increasing transformed volume fraction, *f*, however, the value are quite different. For the as-cast sample, the activation energies are 262 kJ/mol and 126 kJ/mol when transformed volume fraction is 0.2 and 0.8, respectively. The mean value is 181 kJ/mol—while, for the 20% CR sample, *E* are 223 kJ/mol and 110 kJ/mol when transformed volume fraction is 0.2 and 0.8, respectively. The average activation energy is 159 kJ/mol, which is 10% lower than the as-cast sample, indicating that the phase transformation process of the deformed sample is much easier than the as-cast sample.

### 3.4. Kinetic Exponent

The kinetic exponent, also called the Avrami exponent, is a key kinetic parameter that can directly obtain the phase transition mode. The kinetic exponent is generally determined based on the well-known Johnson–Mehl–Avrami (JMA) model [30], which has the following form:

(3) $$f=1-exp\left(-Kt^{n}\right),$$

where *n* is the Avrami exponent, *K* is a rate constant and *t* is the time. In case of phase transformations that are carried out at constant heating rate, the time can be expressed as [31]:

(4) $$t-t_{0}=\frac{T-T_{0}}{\emptyset },$$

where *T*_0_ and *t* are the starting temperature and time of phase transition. Then, the kinetic exponent can be obtained by some mathematical treatment of Equations (3) and (4), which can be expressed as:

(5) $$n=-\frac{\ln\left(-\ln\left(1-f\right)\right)}{\frac{E}{RT}}.$$

Based on Equation (5), we can determine the kinetic expoent *n* from the slope of ln(−ln(1 − *f*)) vs. 1/*T* curve.

For a real phase transformation, *n* will change with the transformed volume fraction. A simple derivative equation of Equation (5) can determine the Avrami exponent as variables, which has been used in many previous published papers [31,32,33]. In this case, *n* is called local Avrami exponent, which can be described as:

(6) $$n=-\frac{\partial \ln\left(-\ln\left(1-f\right)\right)}{\partial \frac{E}{RT}}.$$

**Figure 6:**
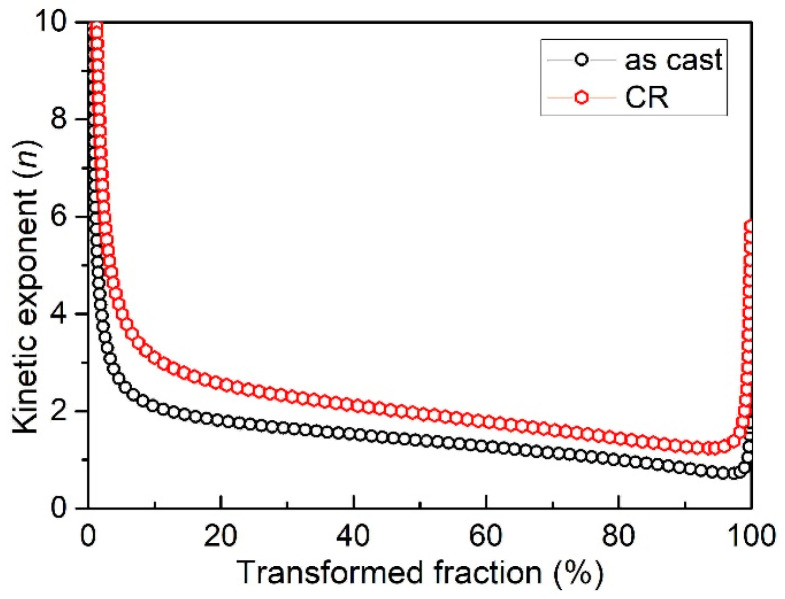
Variation of *n* with phase transformed fraction for as-cast and deformed Al_0.5_CoCrFeNi high-entropy alloy at the heating rate of 10 K/min.

By taken the activation energy at a different transformed volume fraction using Equation (2), Avrami exponent *n* can be calculated by the differential of the curve of E/RT − ln[−ln(1−f)]. Figure 6 represents the kinetic exponent at different transformed volume fractions at the heating rate 10 K/min for the as-cast and CR sample. According to Figure 6, two important pieces of information can be drawn. First, both of the as-cast and deformed samples show three typical stages of transformation: (I) rapid decrease of the kinetic exponent when *f* < 5%, indicating that the nucleation rate of the sample is decreasing at the beginning; (II) slow decrease of the kinetic exponent from 4 to 1.5 during 9% < *f* < 95%, indicating the decreasing nucleation rate, and the growth mode of the nuclei changes from interface controlled to diffusion controlled; (III) strong increase of the kinetic exponent when *f >* 95%, an indication that some new inhomogeneous nucleation occurs inside the sample. Second the kinetic exponent, *n*, is higher for the sample pre-deformed than the as-cast sample. This means that the nucleation rate of the deformed sample is much higher and the growth of the nuclei is much easier than the as-cast sample.

### 3.5. Discussion

**Figure 7:**
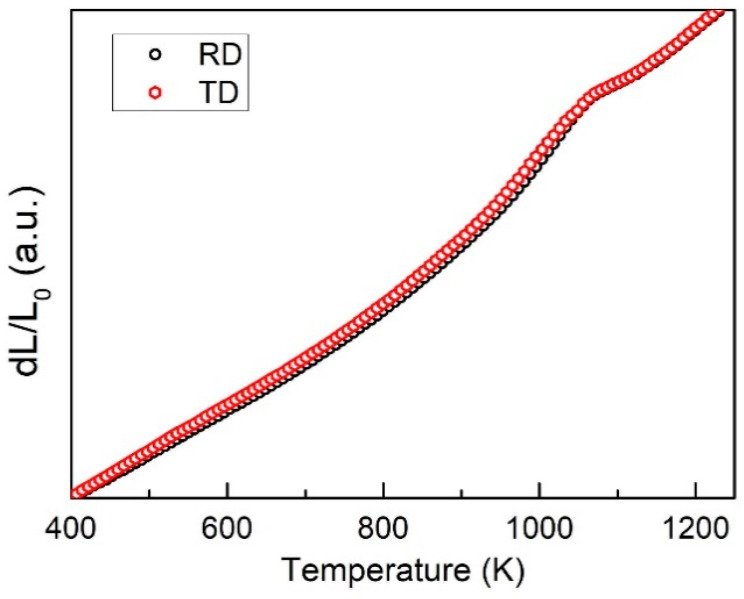
The thermal expansion curves of the samples taken from the rolling direction (RD) and transverse direction (TD) for 20% cold rolled Al_0.5_CoCrFeNi high-entropy alloy measured at the heating rate of 10 K/min.

For the deformed sample like cold rolling, normally, there will be an anisotropic effect due to the stress state during deformation are different at different directions. For the cold rolled sample, the rolling direction (RD) and the transverse direction (TD) always owns different textures, stress and properties. For the above results, all of the analysis is based on the sample that is taken from the RD direction. Figure 7 shows the thermal expansion curves that are both taken from the RD and TD direction of a 20% CR sample. According to the figure, it can be seen that the rolling direction has quite a limited effect on the thermal expansion behavior. The reason may be due to the fact that a 20% reduction is not large enough to generate a big difference, and it can also be found from Figure 1 that the CR sample has a similar microstructure as the as-cast sample. Large differences can be made evident at much larger CR reduction thickness, and the grain will be severely deformed and elongated along the rolling direction [12]. In this case, the thermal expansion behavior could be different for different directions. Thus, it can be concluded that there is no typical anisotropic effect in the 20% CR sample, and, during the solid state phase transformation process, the nucleation and growth process can be treated as homogeneous.

**Figure 8:**
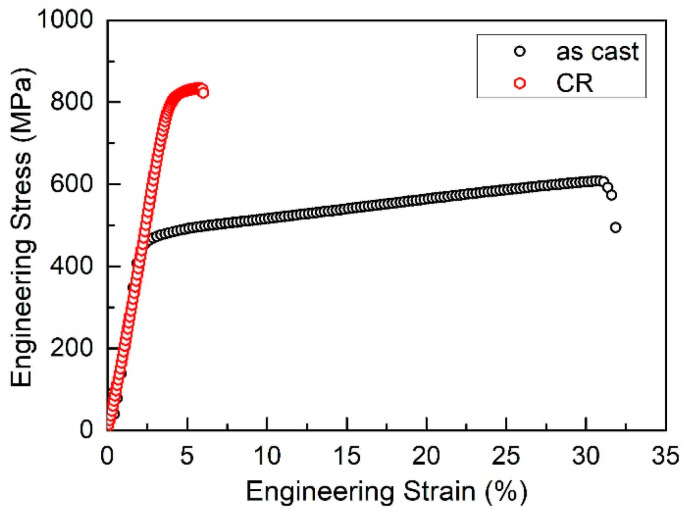
The engineering stress–strain curves of Al_0.5_CoCrFeNi high-entropy alloys at as-cast and 20% CR conditions.

There is not much difference for the microstructure (Figure 1) and phases (Figure 2) between the as-cast and deformed sample; however, according to the *f*–*T* curve, activation energy and kinetic exponent, there is a large difference. The microstructure of Al*_x_*CoCrFeNi (0 ≤ *x* ≤ 2) HEAs has been intensively investigated in different heat treatment conditions [17,18,19,20,21,22,23,24,25]. Different from the thermo-mechanical processing that can affect the transformation pathways in Al_0.3_CoCrFeNi [34], 20% cold rolling cannot alter the phase transition product [12]. Thus, the microstructure and phases exhibit no difference as shown in Figure 1 and Figure 2. However, the deformed sample shows a much faster transformation rate, much lower activation energy and much larger kinetic exponents, an indication of a large different for the transformation kinetics. Figure 8 exhibits the tensile stress–strain curves of Al_0.5_CoCrFeNi HEAs at as-cast and 20% CR conditions. There exists a large difference: the deformed sample shows a much larger stress but lower plasticity. The yield stress increased from 402 MPa (as-cast sample) to 755 MPa (CR sample), while the strain decreased from 33.7% (as-cast sample) to 6.2% (CR sample), indicating a strong strain-hardening effect of the cold rolling. In the present study, due to the small reduction rate of the CR, there is not much difference in the macroscopic microstructure; however, 20% CR is enough to generate a large crystal lattice distortion, and many crystal defects, like vacancy, dislocation and substructure inside the grain. Then, the deformed sample stored a large quantity of energy, which is beneficial for the phase transition during the continuous heating. Thus, because of all the above factors that are generated by cold rolling, the deformed sample shows a much higher transformation rate since all the defects and stored energy inside the sample can promote the nucleation rate and grain growth speed.

## 4. Conclusions

The effects of cold rolling on the phase transformation kinetics of an Al_0.5_CoCrFeNi high-entropy alloy are studied by the thermal expansion method. The transformed volume fraction–temperature curves of as-cast and 20% cold rolling Al_0.5_CoCrFeNi high-entropy alloys are determined by the lever rule method using thermal expansion curves. Using the KAS method, the mean activation energy for as-cast and deformed HEAs calculated are 181 kJ/mol and 159 kJ/mol, respectively. The kinetic exponent is determined using the local Avrami exponent method based on the JMA equation. Both as-cast and deformed samples show the three stages of kinetic exponent variations, and the deformed alloy shows a much larger value compared with the as-cast alloy, indicating a much higher nucleation and growth rate. The reason for the kinetic exponent variations are thought to be related with the deformation induced strain and defects inside the sample that can generate a much higher energy that favors the nucleation and growth process.
